# Function of Platelet Glycosphingolipid Microdomains/Lipid Rafts

**DOI:** 10.3390/ijms21155539

**Published:** 2020-08-02

**Authors:** Keisuke Komatsuya, Kei Kaneko, Kohji Kasahara

**Affiliations:** Laboratory of Biomembrane, Tokyo Metropolitan Institute of Medical Science, Tokyo 156-8506, Japan; komatsuya-ks@igakuken.or.jp (K.K.); ikekoneka@isc.chubu.ac.jp (K.K.)

**Keywords:** lipid rafts, detergent-resistant membrane, heterogeneity, platelets, lysenin

## Abstract

Lipid rafts are dynamic assemblies of glycosphingolipids, sphingomyelin, cholesterol, and specific proteins which are stabilized into platforms involved in the regulation of vital cellular processes. The rafts at the cell surface play important functions in signal transduction. Recent reports have demonstrated that lipid rafts are spatially and compositionally heterogeneous in the single-cell membrane. In this review, we summarize our recent data on living platelets using two specific probes of raft components: lysenin as a probe of sphingomyelin-rich rafts and BCθ as a probe of cholesterol-rich rafts. Sphingomyelin-rich rafts that are spatially and functionally distinct from the cholesterol-rich rafts were found at spreading platelets. Fibrin is translocated to sphingomyelin-rich rafts and platelet sphingomyelin-rich rafts act as platforms where extracellular fibrin and intracellular actomyosin join to promote clot retraction. On the other hand, the collagen receptor glycoprotein VI is known to be translocated to cholesterol-rich rafts during platelet adhesion to collagen. Furthermore, the functional roles of platelet glycosphingolipids and platelet raft-binding proteins including G protein-coupled receptors, stomatin, prohibitin, flotillin, and HflK/C-domain protein family, tetraspanin family, and calcium channels are discussed.

## 1. Platelet Lipid Rafts

The fluid mosaic model has supported our understanding of cellular membranes for a long time. Recent studies suggest that plasma membrane lipids are not homogeneously distributed and that the membranes may contain microdomains or compartments. Glycosphingolipids form microdomains containing cholesterol in the cell membrane. Glycosphingolipid- and cholesterol-rich microdomains are referred to as lipid rafts. Lipid rafts are dynamic assemblies of glycosphingolipids, sphingomyelin, cholesterol, and proteins which are stabilized into platforms involved in the regulation of a number of cellular processes [1]. Lipid rafts are isolated as a detergent-resistant membrane (DRM) fraction by sucrose density gradient centrifugation. Recent studies have demonstrated that lipid rafts are spatially and compositionally heterogeneous in the cell membrane. In migrating T cells, GM3 ganglioside-rich rafts containing a chemokine receptor are present at their leading edge, whereas GM1-rich rafts containing integrin β1 are present at their uropod [2].

In 1996, platelet DRM was shown to be rich in glycoprotein CD36, Src, and Lyn [3]. Platelet rafts are important membrane microdomains in responses such as adhesion and aggregation. The localization of the adhesion receptor glycoprotein (GP)Ib-IX-V complex to lipid rafts is required for platelet adhesion to the vessel wall by binding the von Willebrand factor (vWF) [4,5]. In resting platelets, phosphatidylserine (PS) is asymmetrically restricted to the inner leaflet of the plasma membrane. An increase in intracellular Ca^2+^ concentration during platelet activation can lead to the exposure of PS in the outer leaflet. PS forms a procoagulant binding site for tenase and prothrombinase coagulation complexes. Lipid rafts are required for the release of PS-exposing extracellular vesicles from platelets [6]. Thus, lipid rafts are critical membrane domains in platelet activation processes [7,8]. Interestingly, platelet DRM shifts to a higher density in sucrose gradients upon thrombin receptor activating peptide (TRAP) stimulation [9]. Trace amounts of actin are observed in rafts from resting platelets, but a marked increase in the amount of actin is found in rafts upon platelet stimulation by TRAP. Platelet DRM also shifts to a higher density in sucrose gradients upon adenosine diphosphate (ADP) stimulation [10].

A protease-nicked and biotinylated derivative (BCθ) of perfringolysin O (θ-toxin) binds specifically to cholesterol-rich microdomains of intact cells [11]. In resting platelets, BCθ-positive cholesterol-rich rafts are uniformly distributed on the cell surface. Upon interaction with fibrinogen, BCθ-positive cholesterol-rich rafts accumulate at the tips of filopodia and at the leading edge of spreading platelets [12]. The adhesion-dependent raft aggregation is accompanied by the concentration of the tyrosine kinase c-Src and the tetraspanin CD63 in cholesterol-rich rafts. The perfringolysin O derivative BCθ recognizes a subpopulation (cholesterol-rich rafts) of platelet DRM rafts, suggesting that a heterogeneous population of lipid rafts exists in platelets [11]. However, little is known about raft heterogeneity in platelet membranes.

## 2. Sphingomyelin-Rich Rafts of Platelets

**Figure 1:**
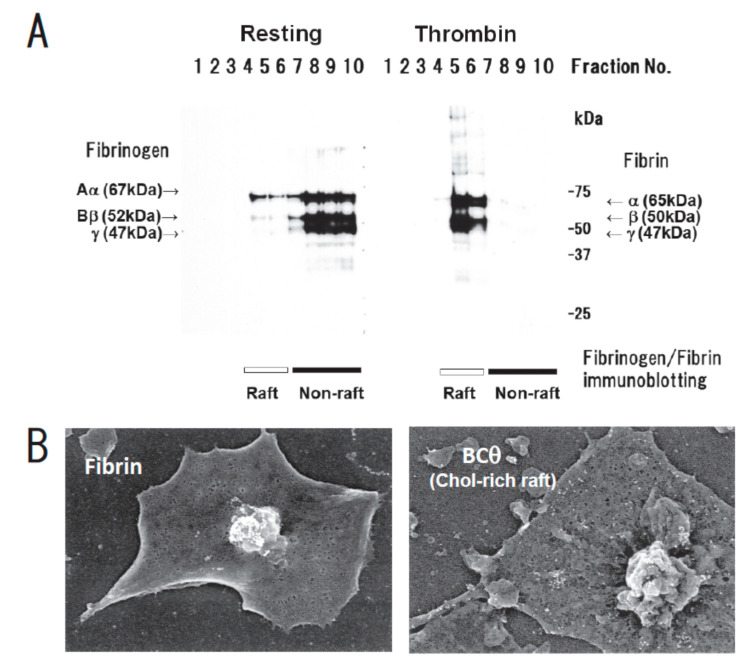
Fibrin translocation to lipid rafts in central region of spreading platelets stimulated with thrombin. (**A**) Sucrose density gradient analysis of washed human platelets. Resting platelets (left) and platelets stimulated for 3 min with 1 U/mL thrombin (right) were lysed in Triton X-100 and then adjusted to 40% sucrose. A sucrose gradient (5–30%) in a volume of 6 mL was layered over the lysate (4 mL) and was centrifuged. Ten fractions were collected from top to bottom after centrifugation and subjected to immunoblotting with an anti-fibrinogen polyclonal antibody. In resting platelets, fibrinogens Aα (67 kDa), Bβ (52 kDa), and γ (47 kDa) were detected in the non-raft fraction (lanes 7–10). In contrast, fibrins α (65 kDa), β (50 kDa), and γ (47 kDa) were detected in the raft fraction (lanes 5,6) of thrombin-stimulated platelets. (**B**) Localization of fibrin (**left panel**) and BCθ-positive cholesterol-rich rafts (**right panel**) of thrombin-stimulated spreading platelets on fibronectin by scanning immunoelectron microscopy. Spreading platelets were incubated with 15 μg/mL BCθ for 30 min followed by glutaraldehyde fixation and immunolabeling with anti-biotin IgG gold. Gold-positive fibrins were localized in the central region of spreading platelet (**left**). In contrast, gold-positive cholesterol-rich rafts were localized uniformly on the membrane (**right**). The study was approved by the institutional ethics committee.

We have been identifying glycosphingolipid-binding proteins [13,14,15,16,17,18,19] and investigated the signal transduction in lipid rafts of platelets [20]. Previously, we reported that clot retraction is mediated by the coagulation factor XIII (FXIII)-dependent fibrin-integrin αIIbβ3-myosin axis in platelet sphingomyelin (SM)-rich membrane rafts [21]. Clot retraction is a process driven by outside-in signaling by the platelet integrin αIIbβ3, resulting in the contraction of the fibrin mesh and the formation of mechanically stable thrombi. To elucidate the function of platelet lipid rafts, we identified DRM-raft-specific proteins from activated platelets. We isolated the DRM raft fraction of platelets treated with thrombin by sucrose gradient centrifugation. Several specific proteins were present in the DRM fraction of thrombin-stimulated platelets. By mass spectrometry, we identified three proteins of 65, 50, and 47 kDa as fibrins α, β, and γ, respectively. These findings were supported by the results of immunoblot analysis using an anti-fibrinogen/fibrin polyclonal antibody. In resting platelets, fibrinogens Aα (67 kDa), Bβ (52 kDa), and γ (47 kDa) were present in the non-raft fraction. In contrast, fibrins α (65 kDa), β (50 kDa), and γ (47 kDa) were exclusively present in the DRM fraction of platelets treated with thrombin (Figure 1A) [21]. Therefore, we investigated the subcellular distribution of fibrin and BCθ-positive cholesterol-rich rafts on thrombin-stimulated spreading platelets by scanning immunoelectron microscopy. Fibrin was localized in the central area of spreading platelet (Figure 1B, left panel). In contrast, BCθ-positive cholesterol-rich rafts were localized evenly on the membrane (Figure 1B, right panel). These observations suggest that fibrin is translocated to platelet rafts other than cholesterol-rich rafts following thrombin stimulation.

**Figure 2:**
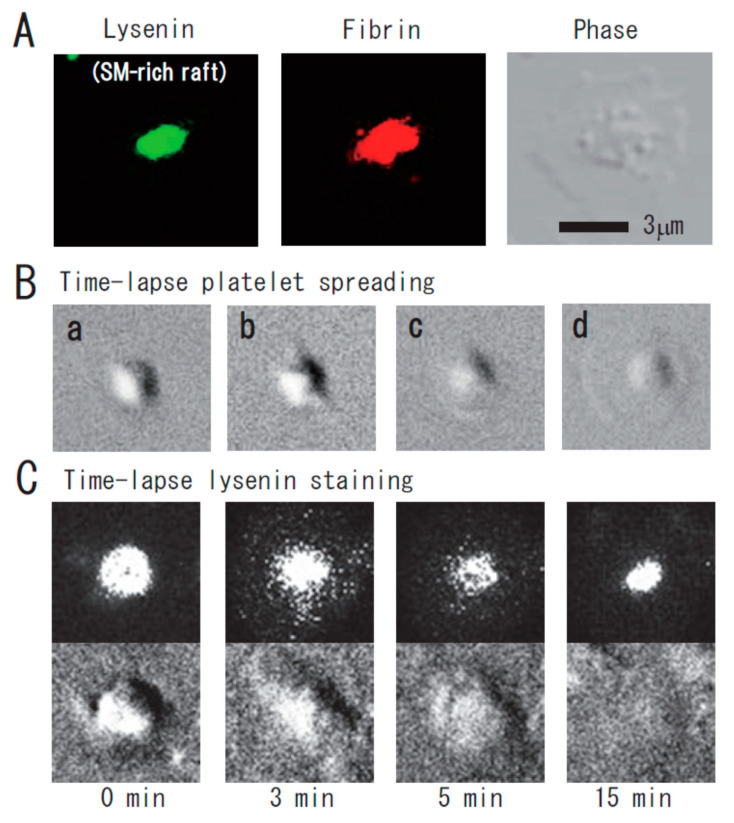
Immunocytochemical colocalization of fibrin with sphingomyelin-rich rafts in central region of spreading platelets stimulated with thrombin. (**A**) Immunocytochemical colocalization of fibrin with sphingomyelin-rich rafts in central region of thrombin-stimulated spreading platelets. Green fluorescent protein (GFP)–lysenin-positive sphingomyelin-rich rafts (**left panel**). Alexa 594-labeled fibrin (**middle panel**). Phase contrast (**right panel**). Scale bar, 3 μm. (**B**) Time-lapse platelet spreading after thrombin stimulation on fibronectin-coated glass strip. (**a**) 0 min; (**b**) 0.2 min, filopodia formation; (**c**) 2 min, spreading; (**d**) 10 min, complete spreading. (**C**) Time-lapse lysenin-positive sphingomyelin-rich raft staining. Washed platelets were incubated with GFP-lysenin for 10 min and then stimulated with 1 U/mL thrombin. The time-lapse fluorescent and DIC images were captured using Olympus LCV110.

**Figure 3:**
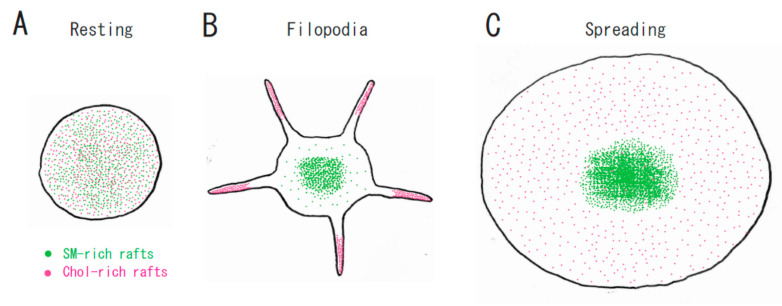
A spatial distinction between SM-rich rafts and cholesterol-rich rafts in platelets. (**A**) In resting platelets, SM-rich rafts (green) and cholesterol-rich rafts (red) are uniformly distributed on the cell surface. No spatial distinction is observed by confocal microscopy. (**B**) Cholesterol-rich rafts accumulate at the tips of filopodia of adhering platelets [12]. SM-rich rafts are mainly localized in the central area of adhering platelets with some distributed in the lamellipodia. (**C**) SM-rich rafts are in the central area of full spreading platelets. Cholesterol-rich rafts were localized evenly on the membrane [21].

Lysenin, the earthworm toxin, is a specific probe of sphingomyelin (SM)-rich rafts in living cells [22,23]. SM is a major component of raft lipids in platelets [9]. Therefore, we investigated the subcellular distribution of SM-rich rafts in spreading platelets. Lysenin-positive SM-rich rafts were localized in the central area of adhering platelets stimulated with thrombin (Figure 2A, left panel). Lysenin-positive SM-rich rafts and fibrin mostly colocalized as a patch in the double-stained the central area of spreading platelets stimulated with thrombin (Figure 2A, middle panel). Next, we investigated the spreading of platelets by time-lapse differential interference contrast (DIC) imaging (Figure 2B) and lysenin staining (Figure 2C). In resting platelets (Figure 2C, 0 min), lysenin-positive SM-rich rafts were uniformly distributed on the cell surface. At an early stage of the spreading of platelets treated with thrombin for 3 min, SM-rich rafts were mainly localized in the central area of adhering platelets with some distributed in the lamellipodia. At a late stage of spreading of platelets treated with thrombin for 15 min, almost all SM-rich rafts were in the central area. Furthermore, we also demonstrated the translocation of myosin to the DRM raft fraction following thrombin stimulation and the colocalization of activated myosin with fibrin in SM-rich rafts of adhering platelets stimulated with thrombin [21]. These observations suggest that SM-rich rafts act as platforms of fibrin-mediated outside-in signaling, leading to clot retraction. To support this idea, the clot retraction of SM-depleted platelets from SM synthase 1 and SM synthase 2 knockout mice was delayed significantly. As a result, we demonstrated that fibrin converted by thrombin translocates immediately into platelet DRM rafts in a coagulation factor XIII (FXIII)-dependent manner. Therefore, we proposed that fibrin is translocated to SM-rich rafts in the presence of FXIII crosslinking activity and that platelet SM-rich rafts act as platforms where extracellular fibrin and intracellular actomyosin join to promote clot retraction [21,24,25]. A spatial distinction between SM-rich rafts and cholesterol-rich rafts in platelets is illustrated (Figure 3).

## 3. Raft Heterogeneity

Platelet DRM shifts to a higher density in sucrose gradients upon platelet activation, suggesting that platelet lipid rafts are dynamic membrane microdomains. Not only actin and fibrin but also small GTPases (Rac, cdc42) and cytoskeleton regulatory proteins (moesin, Arp3, VASP) were detected in the DRM fraction of activated platelets [9]. The possible mechanism of the DRM shift to a higher density in sucrose gradients upon platelet activation presumably involves the high protein-to-lipid ratio [26].

In porcine lung membranes, two distinct types of DRM were obtained after sucrose density gradient centrifugation using Triton X-100. Light DRM contained cerebroside, whereas dense DRM contained Ca^2+^ATPase and the IP3 receptor [27]. In the adult mouse cerebellum, two distinct types of DRM were also obtained after sucrose density gradient centrifugation using Triton X-100. Light DRM contained cerebroside and sulfatide [28]. In B-lymphocytes, two distinct types of DRM were obtained after sucrose density gradient centrifugation using Brij 98. Light DRM contained ganglioside GM1 and MHC II, whereas dense DRM contained ganglioside GM2 and MHC I [29]. These results suggest endocytosis of MHC molecules by distinct lipid rafts. In HEK293T cells, two distinct types of DRM were also obtained after sucrose density gradient centrifugation using sodium carbonate (pH 11). Light DRM contained ganglioside GM1, whereas dense DRM contained cholesterol and flotillins [30]. Therefore, the platelet DRM shifts in sucrose gradients might be due to changes in lipid composition. Lactosylceramide and ganglioside GM3 are the major glycosphingolipids of human platelets [31]. Resting platelets do not express ganglioside GD3. The stimulation of platelets with ADP resulted in the formation of ganglioside GD3 by GD3 synthesis from the GM3 pool [31,32]. The GD3 synthase is CMP-NeuAc:NeuAc α2-3Gal β1-4Glc β1-1′Cer α2,8-sialyltransferase [33]. The stimulation of platelets with thrombin showed an increase in the amount of ganglioside GM3 [34]. The stimulation of platelets with ADP showed a decrease in the amount of cholesterol in the DRM raft fraction [10]. The precise mechanism of DRM shifts to a higher density in sucrose gradients upon platelet activation remains to be elucidated.

## 4. Platelet Glycosphingolipids

Lactosylceramide is the most abundant neutral glycosphingolipid. Its major fatty acids are 20:0, 22:0, 24:0, and 24:1. Ganglioside GM3 is the most abundant acidic glycosphingolipid. The neuraminic acid component was N-acetylneuraminic acid [35,36]. In addition, galactosylceramide [36], sulfatide [37], glucosylceramide [34,38], ganglioside GM1 [39], globotriaosylceramide Gb3 [40], and sialyl-galactosylgloboside [41] are also found in human platelets.

Sulfatide is present on platelet surfaces that bind to adhesive proteins such as vWF, P-selectin, laminin, and thrombospondin [42,43]. Sulfatide is localized as a large cluster towards the center of spreading platelets [44], suggesting that sulfatide-rich rafts may be platforms involved in intracellular signaling. Sulfatide micelles, the sulfatide-binding recombinant malaria circumsporozoite protein (MCSP), and the sulfatide-specific single-chain fragment variable antibody probe PA38 inhibit this adhesion [44,45,46]. The sulfatide antagonist MCSP reverses platelet aggregation induced by ADP, collagen, or TRAP [45]. Sulfatide-deficient mice display an extended lag phase of collagen-induced platelet aggregation [44].

The adaptor protein Disabled-2 (Dab2) as a key regulator of platelet signaling is a sulfatide-binding protein. Its interaction is mediated by two *N*-terminal conserved basic motifs (amino acid residues 24–29 and 49–54) with a dissociation constant Kd of 0.6 μM [47]. Dab2 is present in the cytoplasm and α-granules of platelets and is released from the platelets in response to platelet activation. Dab2 interacts with the cytoplasmic tail of the integrin αIIbβ3 and regulates inside-out signaling [48]. On the other hand, Dab2 released from α-granules inhibit platelet aggregation by competing with fibrinogen for binding to the integrin αIIbβ3, an interaction that is modulated by Dab2 binding to sulfatide at the outer leaflet of the plasma membrane. The Dab2 sulfatide-binding motif peptide can prevent sulfatide-induced platelet aggregation [49,50]. The bleeding time is prolonged and thrombus formation is impaired in Dab2-deficient mice. Dab2-deficient platelets elicited a selective defect in platelet aggregation and spreading on fibrinogen by thrombin stimulation [51].

Sulfatide on the platelet surface interacts with a blood coagulation factor, playing a major role in hemostasis. Blood coagulation cascade has two pathways: intrinsic pathway and extrinsic pathway. Coagulation factor XII is a plasma serine protease that initiates the intrinsic pathway of blood coagulation upon contact with anionic surfaces, such as sulfatide on the plasma membrane. Annexins (ANXs) are implicated in the regulation of blood coagulation reactions by binding to sulfatide [52]. ANXA3, ANXA4, and ANXA5 inhibit sulfatide-induced plasma coagulation. ANXA4 inhibits sulfatide-induced autoactivation of Factor XII to Factor XIIa and the conversion of its natural substrate Factor XI to Factor XIa [53].

Ganglioside GD3 is rapidly expressed on the platelet surface following platelet activation and internalized to the cytoskeleton where it transiently associates first with the Src family kinase Lyn then with the Fc receptor gamma chain [32]. The binding of bacterial cells to human platelets contributes to the pathogenesis of infective endocarditis. Platelet binding by *Streptococcus mitis* strain SF100 is mediated by two surface proteins, PblA and PblB. α2-8-linked sialic acid residues on platelet membrane ganglioside GD3 are the primary targets for PblA/PblB-mediated binding to human platelets. [54].

Globotriaosylceramide Gb3 is a functional receptor of the Shiga toxin [40]. Shiga toxin is the principal virulence factor of enterohemorrhagic *Escherichia coli*. Thrombocytopenia caused by platelet consumption in thrombi is a primary symptom of hemolytic uremic syndrome associated with Shiga toxin. Shiga toxin1 and its B (binding) subunit bind to platelets, leading to fibrinogen binding and platelet aggregation [55]. The possible existence of glycosphingolipid-specific rafts, such as sulfatide-rich rafts, remains to be explored.

## 5. Platelet Raft-Binding Proteins

Platelet rafts function as dynamic membrane microdomains for the attachment of various proteins such as adhesion molecules, receptors, signaling molecules, adaptor proteins, and effector proteins (Table 1).

### 5.1. Protein S-Palmitoylation: Lipid Raft Targeting Modification

S-palmitoylation is a posttranslational modification catalyzed by palmitoyl acyltransferases from the zincfinger and Asp–His–His–Cys domain-containing (DHHC) enzyme family. It is involved in the attachment of the saturated palmitoyl acyl chain (C16:0) delivered by palmitoyl-CoA to a cysteine residue [79,80,81]. DHHC4 and DHHC5 facilitate fatty acid uptake by palmitoylating and targeting CD36 to the plasma membrane [82]. DHHC5 palmitoylates flotillin-2 in neuronal cells [83]. DHHC2 affects palmitoylation, and functions of tetraspanins CD9 and CD151 [84]. The enzymatic removal of S-acyl modifications in mammalian cells is catalyzed by acyl protein thioesterase (APT) and APT can remove palmitate groups from palmitoylated proteins [80]. Two protein palmitoyl thioesterases (PPTs) have been described as being capable of catalyzing the removal of fatty acids from proteins, in other words, acyl protein thioesterase 1 (APT1), and palmitoyl protein thioesterase 1 (PPT1). APT1 is reported to depalmitoylate the alpha subunit of G proteins and LAT in vitro. APT1 is itself palmitoylated and contain a hydrophobic pocket to accept palmitoylated substrates.

Protein palmitoylation is a dynamic modification that regulates the lipid raft targeting of proteins [85]. The basic forces driving raft formation are lipid interactions. The saturated acyl chains and high acyl chain melting temperatures of glycosphingolipids mediate glycosphingolipid clustering in combination with cholesterol, which has the properties of a “liquid-ordered phase.” In contrast, most phospholipids have unsaturated acyl chains, low melting temperatures, and the properties of a liquid-disordered phase. Lipid rafts are considered to exist as phase-separated domains. The linkage of membrane proteins to saturated acyl chains by palmitoylation is considered to facilitate the translocation of these proteins to lipid rafts.

Platelet raft marker proteins are characterized by multiple S-palmitoylations. For example, palmitoylation occurs on the two N-terminal and two C-terminal cysteines, in human CD36 corresponding to cysteine residues 3, 7, 464, and 468 [86]. Both cysteine pairs are intracellular and adjacent to transmembrane segments. LAT contains two palmitoylated cytoplasmic Cys residues adjacent to its transmembrane domain, Cys 26 and Cys 29 [81]. Caveolin-1 is S-palmitoylated on its three cysteine residues (Cys 133, Cys 143, and Cys 156). [87] Using a proteomic approach, 215 palmitoylated platelet proteins are identified [88]. Palmitoylated platelet-raft-binding proteins are indicated in Table 1.

### 5.2. G protein-Coupled Receptors (P2Y1, P2Y12, CXCR4)

Platelet activation by several agonists such as collagen, ADP, and thrombin is followed by platelet granule release, integrin αIIbβ3 activation, aggregation, and thrombus formation. All these processes are triggered by an increase in cytosolic Ca^2+^ concentration ((Ca^2+^)i). Ca^2+^, diacylglycerol-regulated guanine nucleotide exchange factor I, and protein kinase C have been shown to be critical elements that link increased (Ca^2+^) to platelet secretion and integrin αIIbβ3 activation (inside-out signaling). ADP induces multiple platelet responses via seven transmembrane G protein-coupled receptors, P2Y1, and P2Y12. Lipid raft integrity is required for the P2Y1 and P2Y12 signaling pathways. P2Y1 is translocated to the DRM raft fraction by in vitro stimulation with ADP [10]. Importantly, in vivo oral administration to rats with clopidogrel, a P2Y12 antagonist, induces disruption of P2Y12 oligomers and their partition removal from lipid rafts [89].

Platelets are a source of chemokine stromal cell-derived factor-1α (SDF-1α), which is stored in α-granules. Platelet-derived SDF-1α modulates paracrine mechanisms such as chemotaxis [90]. Platelet-derived SDF-1α is also an autocrine activator of platelets through its receptor CXCR4 [91,92,93,94]. SDF-1α-induced platelet aggregation in inhibited by the pertussis toxin, suggesting that its effect is mediated by a pertussis-toxin-sensitive G protein such as Gαi. SDF-1α induces platelet aggregation via phosphatidylinositol 3 kinase (PI3K)/Akt signaling pathway [20]. Furthermore, SDF-1α-induced platelet aggregation and Akt phosphorylation are inhibited by pretreatment with the raft-disrupting agent methyl-β-cyclodextrin. Sucrose density gradient analysis shows that CXCR4 (35%), the heterotrimeric G proteins Gαi-1 (93%), Gαi-2 (91%), and Gβ (50%) and PI3Kβ (4%), and Akt2 (4.5%) are localized in the DRM raft fraction. Gαi-1 and Gαi-2 are S-palmitoylated on a cysteine residue (Cys3). SDF-1α is highly expressed in atherosclerotic plaques [95], suggesting that platelet aggregation by SDF-1α/CXCR4 axis contributes to the pathologies such as atherosclerosis. Surface expression of SDF-1α on platelets is a biomarker in ischemic events [90]. The SDF-1α expression level on platelets is elevated in patients with acute myocardial infarction [96].

### 5.3. Stomatin, Prohibitin, Flotillin, and HflK/C (SPFH)-Domain Protein Family

#### 5.3.1. Flotillin

Flotillins are raft-associated integral membrane proteins and belong to the SPFH superfamily [97]. Flotillins bind the inner leaflet of a plasma membrane raft and serve as scaffolds facilitating the assembly of multiprotein complexes. Flotillin-1 and flotillin-2 have the same domain architecture, comprising two domains: the *N*-terminal SPFH domain and the *C*-terminal flotillin domain [98]. The SPFH and flotillin domains mediate inner membrane binding and oligomerization of flotillins, respectively. The membrane association of flotillins is determined by the acyl chain(s) attached and the interaction of protein hydrophobic regions with the cytosolic leaflet of membranes. Flotillin-1 is S-palmitoylated on Cys34 located within the first hydrophobic stretch (amino acids 10–36). Flotillin-2 is N-myristoylated on Gly2 and S-palmitoylated on three cysteine residues; Cys4, Cys19, and Cys20, which are located in the first hydrophobic region (amino acids 14–35). The second hydrophobic region locates in the middle part of the SPFH domain (amino acids 134–150/151). The binding of cholesterol by flotillins is mediated by the cholesterol recognition/interaction amino acid (CRAC) motif(s) located within the SPFH domain (amino acids 117–124 in flotillin-1; 120–127 and 157–169 in flotillin-2).

Platelets store sphingosine-1-phosphate (S1P) abundantly and release this bioactive lipid extracellularly upon stimulation [99,100]. S1P induces platelet shape change and aggregation reactions and stimulates vascular endothelial cell spreading and migration [101]. Platelet-derived S1P plays an important role in vascular biology. S1P is synthetized from sphingosine by sphingosine kinases. Recently, flotillin-1 and flotillin-2 have been shown to recruit sphingosine to lipid rafts and maintain cellular S1P levels [102]. Sphingosine binding is mediated by the SPFH domain of flotillins, but the exact identities of the hydrophobic sequences of the flotillins involved are not known. Flotillins also interact with numerous signaling proteins such as receptors, protein kinases, G proteins, and adaptors [98]. Therefore, flotillin-based microdomains can serve as platforms mediating the formation of multiprotein complexes and transmembrane signal transduction at the plasma membrane.

#### 5.3.2. Stomatin

Stomatin is a raft-associated integral membrane protein and belongs to the SPFH superfamily [103]. Stomatin is composed of the *N*-terminal 24-residue basic domain, hydrophobic intramembrane domain (residues 26–54), cholesterol recognition/interaction amino acid consensus (CRAC, residues 55–68), SPFH domain (residues 57–256), coiled-coil domain, oligomerization and lipid-raft-association domain (ORA, residues 263–273), and C-terminal domain. Stomatin is S-palmitoylated on Cys30 and Cys87. The α-helical segments of stomatin flexibly move along with the membrane surface, with such movement potentially leading to membrane bending via lipid raft clustering through the formation of homo-oligomeric complexes of SPFH-domain proteins [97]. Stomatin is localized at the platelet α-granular membrane. The lipid-raft marker proteins flotillin-1 and flotillin-2 are present in the plasma membrane but excluded from α-granules. The activation of platelets by thrombin leads to translocation of stomatin to the plasma membrane [59]. Lipid raft-associated stomatin enhances cell fusion. With its unique molecular topology, stomatin forms molecular assemblies within lipid rafts, and promotes membrane fusion by modulating fusogenic protein engagement [104]. During platelet activation, the α-granular membrane undergoes fusion with the platelet plasma membrane and granular secretion. Stomatin may have a role in the α-granular membrane fusion.

#### 5.3.3. Prohibitin

Prohibitin is also a raft-associated integral membrane protein and belongs to the SPFH superfamily [105]. Prohibitins, comprising the two homologous members PHB1 and PHB2, are ubiquitously expressed and highly conserved. Prohibitin is composed of the *N*-terminal hydrophobic stretch, SPFH domain, and coiled-coil domain. Prohibitin is S-palmitoylated on Cys69 [106]. Prohibitins are distributed in lipid rafts, as determined by sucrose density centrifugation. In addition, prohibitins are associated with protease-activated receptor 1 (PAR1). Platelet aggregation, integrin αIIbβ3 activation, granular secretion, and calcium mobilization stimulated by low-concentration thrombin are reduced by the blockade of prohibitins with anti-prohibitin antibody [72]. Prohibitins are involved in PAR1-mediated platelet aggregation.

### 5.4. Tetraspanin Family

Tetraspanins are a superfamily of cell-surface glycoproteins that are characterized by four transmembrane domains, intracellular N- and C-termini, and conserved sequence motifs within the larger of two extracellular regions. Tetraspanins are considered to function by self-associating to form a novel type of membrane microdomain, “tetraspanin-enriched microdomains (TEMs)”. TEMs are physically and functionally distinct from lipid rafts [107]. However, gangliosides are a membrane component of TEM [108] and are involved in tetraspanin–partner interactions, as determined from the finding that the depletion of gangliosides affects the interaction between CD82 and its partners [109], suggesting that gangliosides play a critical role in the organization of TEMs. Therefore, TEMs are considered to be a subset of glycosphingolipid microdomains [110].

Tetraspanins are a family of 33 membrane proteins in humans. More than ten tetraspanins (CD9, CD63, CD81, CD82, CD151, and Tspan 2, Tspan 9, Tspan 14, Tspan 15, Tspan 18, Tspan 32, and Tspan 33) are identified in platelets by flow cytometry and proteomics. The relative expression ratio of tetraspanins CD9, CD151, Tspan9, and CD63 (listed in order of their abundance in human platelets) have been estimated at 50:7:3:1.

#### 5.4.1. CD9

CD9 is found to be expressed at approximately 50,000 copies per platelet [111]. CD9 is a negative regulator on platelets, because the fibrinogen binding of integrin αIIbβ3 in response to platelet agonists is found to be mildly enhanced in CD9-deficient platelets, suggesting that CD9 limits the inside-out activation of this integrin [112]. CD9 is S-palmitoylated on six cysteine residues (Cys9, Cys78, Cys79, Cys87, Cys218, and Cys219), which are located in four internal juxta membrane regions [113].

#### 5.4.2. CD151

CD151-deficient platelets exhibited impaired “outside-in” integrin αIIbβ3 signaling with defective platelet aggregation by the protease-activated receptor 4 (PAR4) agonist peptides, collagen, and ADP; impaired platelet spreading on fibrinogen; and delayed kinetics of clot retraction in vitro [114]. Furthermore, tail bleeding assay shows longer bleeding times, leading to the three-fold loss of blood and a seven-fold increase in the incidence of rebleeding [115]. CD151 is S-palmitoylated on six cysteine residues (Cys 11,15,79,80, 242, and 243). The association of a palmitoylation-deficient CD151 with CD81 and CD63 is markedly attenuated, but the interaction of the α3β1-CD151 complex with phosphatidylinositol 4-kinase was not affected [116].

#### 5.4.3. CD63

In resting platelets, CD63 is localized on the membranes of α-granules and dense granules. Following platelet activation and granule exocytosis, CD63 is expressed on the plasma membrane and colocalizes with the αIIbβ3-CD9 complex. CD63-deficient platelets show slightly enhanced in vitro aggregation responses, but they do not affect thrombus formation in vivo [117]. Palmitoylation levels of CD63 and CD9 increase following thrombin activation.

#### 5.4.4. Tspan32

Tspan32(TSSC6)-deficient platelets exhibit impaired clot retraction, platelet aggregation at lower doses of PAR4, and collagen and platelet spreading on fibrinogen. Tspan32-deficient mice exhibit longer bleeding times and an increase in rebleeding, as shown by tail bleeding assay [118].

#### 5.4.5. CD82

CD82-deficient platelets display enhanced integrin αIIbβ3 surface expression, adhesion, tyrosine kinase signaling on fibrinogen, and clot retraction. CD82-deficient mice exhibit reduced bleed times in vivo [119].

A major problem in tetraspanin research is how to determine whether a particular phenotype is due to a specific effect on tetraspanin. CD151 and Tspan32 are direct binding partners of αIIbβ3 and might enhance outside-in signaling by recruiting specific signaling proteins in a subset of glycosphingolipid microdomains.

### 5.5. Calcium Channels (Orai 1, STIM, TRPC)

Platelet activation and aggregation depend on the increase in (Ca^2+^)i resulting from intracellular Ca^2+^ release followed by store-operated Ca^2+^ entry (SOCE) through Ca^2+^ release-activated channels [120]. SOCE is accomplished by the pore forming unit Orai and its regulator the stromal interaction molecule (STIM). STIM1 is a transmembrane protein essential for the activation of SOCE, a major Ca^2+^ influx mechanism. STIM1 is localized in the endoplasmic reticulum, communicating the Ca^2+^ concentration in the stores to plasma membrane channels. Lipid rafts are required for the inactivation of SOCE by extracellular Ca^2+^ mediated by the interaction between plasma-membrane-located STIM1 and Orai1 [70]. Orai1 is a novel candidate of the platelet palmitoylome [88].

Orai1 trafficking to the cell surface is impaired in Tspan18-deficient platelets, resulting in impaired Ca^2+^ signaling. Tspan18 may regulate the Ca^2+^ channel function of Orai1 at the cell surface by promoting its clustering [121]. A reduction in the rate of release and a maximal Ca^2+^ increase are observed in Tspan18-deficient platelets. Defective aggregation of Tspan18-deficient platelets is observed in response to a collagen-related peptide at an intermediate concentration. Tspan18-deficient platelet spreading is impaired on a collagen-related peptide but normal on fibrinogen.

Another family of plasma membrane Ca^2+^ channels, the transient receptor potential canonical (TRPC) channels, also contributes to sustained (Ca^2+^)i elevation. TRPC1, TRPC4, and TRPC5 form a heteromultimer associated with platelet lipid raft domains, whereas TRPC3 and TRPC6 associate independently of lipid rafts [76]. TRPC5 is S-palmitoylated on Cys 181 in an intracellular loop [122].

## Figures and Tables

**Table 1:** Platelet raft-binding proteins.

Molecules	Function	Localization in Rafts	Moves into Rafts	Palmitoylation	Ref.
Actin	Cytoskeleton	+			[9]
ACV/VI	Adenylyl cyclase		PGI2	〇	[56]
Akt2	Ser/Thr kinase	+			[20]
Arp3	Actin nucleator		TRAP		[9]
Caveolin-1	Integral scaffolding protein	++		〇	[57]
CD9	Tetraspanin	++		〇	[58]
CD36	Scavenger receptor	+++		〇	[59]
CD63	Tetraspanin	++		〇	[58]
Cdc42	Small G protein		TRAP	〇	[9]
CLEC-2	Podoplanin receptor		Rhodocytin		[60]
c-Src	Tyr kinase	++			[12]
CXCR4	Chemokine receptor	+			[20]
Estrogen receptor	Hormone receptor		Estradiol		[61]
Factor XI	Plasma thromboplastin	++			[62]
Factor XIII	Transglutaminase		Thrombin		[21]
Fc receptor g	Immunoglobulin G receptor	++			[63]
Fibrin	Major component of blood clot		Thrombin		[21]
Flotillin-1	SPFH-domain scaffolding protein	+++		〇	[59]
Flotillin-2	SPFH-domain scaffolding protein	+++		〇	[64]
Gia	Trimeric G protein	++		〇	[65]
GLUT-3	Glucose transporter	++			[59]
GP130	IL6 receptor	++			[66]
GPIb/IX/V	vWF receptor		vWF	〇	[4]
GPVI	Collagen receptor		Collagen		[67]
Integrin aIIbb3	Fibrinogen receptor	+			[21]
LAT	Linker for activation of T cells	+++		〇	[68]
Lyn	Tyr kinase	++		〇	[69]
Moesin	ERM family		TRAP		[9]
Myosin	Cytoskeleton		Thrombin		[21]
Orai1	Store-operated Ca^2+^ entry	++			[70]
P2X1	ATP receptor	++			[64]
P2Y1	ADP receptor		ADP		[10]
P2Y12	ADP receptor		ADP		[10]
PECAM-1	Adhesion molecule	++		〇	[63]
PI3Kb	Phosphatidylinositol 3-kinase	+			[20]
PI4K55	Phosphatidylinositol 4-kinase	++		〇	[58]
PKA-I	Ser/Thr kinase		PGI2		[56]
PP1c	Protein phosphatase		Thrombin		[71]
PP2Ac	Protein phosphatase		Thrombin		[71]
Prohibitin	SPFH-domain scaffolding protein	++		〇	[72]
PrPc	Prion	+			[73]
Pyk2	Tyr kinase	++			[61]
Rap2b	Small G protein	++		〇	[74]
STIM1	Store-operated Ca^2+^ entry	++			[70]
Stomatin	SPFH-domain scaffolding protein	++		〇	[59]
TIIICBP	Collagen receptor	++			[75]
TRPC1,4,5	Store-operated Ca^2+^ entry	++		〇	[76]
TXA2 receptor	Prostanoid receptor	++		〇	[77]
VASP	Actin filament elongation		TRAP		[9]
vWF	Molecular glue of platelet plug	++			[4]
14-3-3ζ	pSer/pThr binding protein		Cold shock		[78]

Ratio (localization in rafts/non-rafts) +: low, ++: medium, +++: high.

## Abbreviations

ADP	adenosine diphosphate
ANX	annexin
APT	acyl protein thioesterase
BCθ	biotinylated derivative of perfringolysin O
CRAC	cholesterol recognition/interaction amino acid motif
Dab2	disabled-2
DHHC	Asp-His-His-Cys domain
DIC	differential interference contrast
DRM	detergent-resistant membrane
FXIII	coagulation factor XIII
GFP	green fluorescent protein
GP	glycoprotein
MCSP	malaria circumsporozoite protein
PAR1	protease-activated receptor 1
PAR4	protease-activated receptor 4
PGI2	prostaglandin I2
PI3K	phosphatidylinositol 3 kinase
PPT	palmitoyl protein thioesterase
PS	phosphatidylserine
S1P	sphingosine-1-phosphate
SDF-1α	chemokine stromal cell-derived factor-1α
SOCE	store-operated Ca2+ entry
SM	sphingomyelin
SPFH domain	stomatin, prohibitin, flotillin, and HflK/C
STIM	stromal interaction molecule
TEM	tetraspanin-enriched microdomain
TRAP	thrombin receptor activating peptide
TRPC	transient receptor potential canonical
TXA2	stromal interaction molecule
vWF	von Willebrand factor
